# Experiences, opinions and current policies on users’ choice and change of the allocated primary mental health professional: a survey among directors of community mental health centers in the Emilia-Romagna region, Italy

**DOI:** 10.1186/s13033-020-00373-8

**Published:** 2020-05-30

**Authors:** G. Rioli, S. Ferrari, C. Henderson, G. M. Galeazzi

**Affiliations:** 1 Department of Biomedical, Metabolic and Neural Sciences, University of Modena & Reggio Emilia, Via Giuseppe Campi, 287, 41125 Modena, Italy; 2 Clinical and Experimental Medicine PhD Program, University of Modena and Reggio Emilia, Via Giuseppe Campi, 287, 41125 Modena, Italy; 3 Department of Mental Health and Drug Abuse, AUSL Reggio Emilia, viale Amendola 2, 42122 Reggio Emilia, Italy; 4 Centre for Neuroscience and Neurotechnology, University of Modena & Reggio Emilia, Via Giuseppe Campi, 287, 41125 Modena, Italy; 5 Health Service and Population Research Department, King’s College London, Institute of Psychiatry, Psychology and Neuroscience, London, SE5 8AF UK

**Keywords:** Recovery, Choice, Service users, Primary mental health professional, Community mental health center, Emilia-Romagna, Social psychiatry, Quality of care

## Abstract

**Background:**

The subject of how the initial allocation of the primary mental health professional (PMHP) in community mental health services is made and the frequency and management of users’ requests to choose and/or change their allocated PMHPs has been scarcely investigated. The present paper is aimed at exploring the experiences and opinions of directors of community mental health centers (CMHC) on this topic.

**Methods:**

A cross-sectional survey was conducted. Electronic ad hoc questionnaires with both multiple choice and open-ended questions were e-mailed to the institutional addresses of CMHC directors in the Emilia-Romagna Region (Northern Italy) with the consent of their heads of department and the Ethical Committee. Quantitative data were analysed by means of Microsoft Excel software and STATA 14.2 (College Station, TX), while the qualitative analysis was performed using the Nvivo12 software.

**Results:**

Twenty-eight questionnaires were collected (response rate: 71.8%) that were equally distributed between males and females. For the initial PMHP allocation, casual allocation by “fixed-rota” was commonly performed (39.3%). Moreover, hope for a change of prescription by a different psychiatrist was the most frequent reason for users’ requests to change their PMHP. In two mental health departments only (Parma and Bologna), written guidelines to manage users’ requests of change of PMHP were available. In this context, most participants classified the explored topics as relevant and believed that written policies, especially if shared with users, could be useful.

**Conclusions:**

In Emilia-Romagna CMHCs, neither users nor professionals were generally involved in the initial choice of the PMHP. Further national-level studies should be conducted in order to confirm this finding. Additionally, written and shared guidelines for managing users’ request to choose/change their PHMP may be useful.

## Background

In recent decades, medical care and decision-making have shifted from paternalistic to more collaborative and shared models [1–3], promoting patients’ autonomy—one of the four main principles of biomedical ethics [4]—and improving quality of care [5].

According to this paradigm shift, recovery-oriented mental health practice and service delivery affirm users’ right to exercise self-determination, make decisions and be involved in the co-construction of their pathways of care [6–8]. One specific application of such innovative principles would be the involvement of users in decisions about the initial allocation or subsequent changes of the primary mental health professional (PMHP) in community mental health centers (CMHCs). In this context, this paper explores the current practice in Italy for allocating service users to their PMHP (who are in most cases psychiatrists [9]) at intake and the ways by which requests for change of the PMHP by users are dealt with.

The general subject in terms of choice and allocation of treating professionals has been addressed in various countries. In the UK, policy documents generally support the view that users have the right to choose their primary mental healthcare provider and clinical team for out-patient treatment in the National Health Service [10, 11]. Similarly, in Sweden, recent reforms have encouraged the exercise of patient’s choice in outpatient settings—both general practice and other specialities, including mental health [12, 13]. Additionally, according to the Australian Government’s Department of Health, mental health users have the right to “have their wishes respected and taken into account” and the right to “have their age, social, economic, cultural/geographical background and spiritual preferences” as well as their “sexual orientation, gender and gender identity taken into consideration in their treatment, support and care” [14]. These topics have also gained relevance in the planning of mental health services in New Zealand, USA and Canada [15]. The involvement of professionals conducting an intake followed by assigning PMHPs on the basis of the practitioner’s skill with the diagnosis is common in the US [16, 17]. For example, with the “Mental Health Intake Form”—a self-reported questionnaire with a current symptoms’ checklist and treatment goals—service users are able to introduce themselves to mental health professionals before any consultation. Therefore, based on the main issues and concerns relating to mental health and their preferences, users can be allocated the most suitable PMHP [18]. The importance of involving service users and families preferences in every step of their treatment plan, certainly including the choice of provider, is also one of the tenets of the System of Care values and philosophy, originally proposed by Stroul and Friedman in 1986 [19] in order to guide psychosocial interventions for severely disturbed children and youth, a framework still very influential in the discourse about mental health service organization. However, users’ choice of mental health professional appears problematic and is only partially applied in real practice [20].

Meanwhile, in Italy, despite the presence of a long tradition of community-centred provision of mental health [21], users generally cannot choose their PMHP in CMHCs [22]. A preliminary scoping review [23] showed that little research is available on these topics. According to this review, users would prefer to be allowed to express their preferences in terms of choosing their own PMHP [24]. Further, a better user-provider matching in age, gender and ethnic and linguistic background is sometimes considered important, but studies are few and far from conclusive.

Furthermore, a qualitative study that was recently conducted by the authors of this study involving users, caregivers and PMHPs in the Modena area (Northern Italy) [25] found that neither users nor professionals were generally involved in the initial choice of the PMHP and that no official written guideline on these topics was available in the investigated area.

To our knowledge, no study, neither in Italy nor elsewhere, has systematically investigated opinions and experiences on these topics among directors of CMHCs.

Thus, the aim of this paper is to survey the experiences and opinions of directors of CMHCs on topics including initial allocation as well as users’ choices and their requests for change of PMHP; any policy or guideline available on these topics was also enquired on.

## Methods

### Study design

The authors conducted a quantitative cross-sectional survey with qualitative components, through an electronic questionnaire, aiming to investigate the experiences and opinions of directors of CMHCs on the topic of users’ choices and their requests to change the allocated PMHP.

Subsequently, a thematic content analysis of the qualitative component of the survey was carried out [26].

### Setting

The research was implemented in the Emilia-Romagna region of Northern Italy (catchment area: 4.460.580 inhabitants, source: ISTAT, 30.11.2018). In Emilia-Romagna, there are eight mental health departments (MHD: Piacenza, Ferrara, Modena, Reggio Emilia, Romagna, Imola, Parma, Bologna), including 39 CMHCs, with a regional incidence rate of 95.4 new users per 10,000 inhabitants and a prevalence rate of 213.2 users per 10,000 inhabitants [27]. As of 1st January 2018, there were 535,974 foreigners living in Emilia-Romagna, representing 12.0% of the resident population and 8% of mental health service users. Among the foreigners, 167 different nationalities were represented, mostly from North Africa and Eastern European countries. The most frequent were Morocco (16.9% of the total non-Italians), Romania (14.1%) and Albania (11.3%) [28].

### Participant selection

All CHMC Directors of the eight Emilia-Romagna MHDs were invited to participate in the research, without any exclusion.

### Qualitative and quantitative questionnaire surveys

No validated instruments are currently available to explore the topic of interest; hence, the electronic questionnaire used to collect data in the present study was designed ad hoc by researchers (GR and GMG). It consisted of 15 questions, 10 of which were closed, and the remaining five were open-ended. At the beginning, the questionnaire included clear and concise instructions for respondents. A final question enquiring on any existing guidelines or policies in use at their CMHC on the topics of interest was also included.

The construction of the questionnaire entailed several phases: definition of the content areas to be explored with the questionnaire; formulation of related questions or items; and arrangement of questions in a logical order. Questions progressed from the least sensitive to the most sensitive, from the factual and behavioural to the cognitive and from the more general to the more specific in order to ensure that the answer to a question was not influenced by previous questions. The language and technical terminology used were based on the basis of the homogeneous high-level education of the surveyed population.

The questionnaire was pilot tested among six PMHPs and CMHC directors of the Province of Modena. Feedback from such piloting was implemented in the original questionnaire to develop the final version used in the study (available on request from the corresponding author).

### Procedure

On March 2019, an initial e-mail describing the study and its rationale was sent to the institutional e-mail addresses of all MHD Heads. In this e-mail, the researchers introduced themselves briefly and provided information on the research project, concisely describing the topics of the questionnaires and the modality to fill them.

Secondly, with the consent of all MHD Heads, an e-mail along with the electronic questionnaire was sent to the institutional e-mail of each of the 39 CMHC Directors of the Emilia-Romagna Region in order to explore their attitudes, experiences, opinions and information on the research topics.

### Research team and reflexivity

The research electronic questionnaire was e-mailed by GR, a final-year resident in psychiatry at the University of Modena and Reggio Emilia at the time of the study. Questionnaires were collected and analysed by GR. GMG and SF, associate professors in psychiatry at the University of Modena and Reggio Emilia at the time of the research; they also supervised the general research methodology and contributed to the analysis of data.

### Data collection

Each participant was asked to fill out a research questionnaire recording the experiences and opinions on the investigated topics and prompting further feedback. Participants were asked to return the filled-in questionnaires to the institutional e-mail of one researcher (GR). In case of any doubts or need for clarification about the research and/or the questionnaires, participants could email the researchers (GR and GMG).

### Data analysis

Questionnaires were collected in an electronic database and analysed independently by two researchers (GR, GMG).

As far as quantitative data was concerned, dichotomous, numerical and categorical answers were analysed by means of statistical descriptive methods: percentage, frequencies and means through the STATA 14.2 software (College Station, Texas) and Microsoft Excel. Further, the Nvivo 12 software was used to analyse qualitative data by performing a thematic content analysis and developing a hierarchical code system a posteriori (derived from the data).

The dataset on which the conclusions of the paper are based is available for readers as supplementary material from the corresponding author.

### Ethics

This research was approved by the local ethics committee. All Emilia-Romagna MHD heads and CMHC directors agreed to the research protocol and gave their consent to participation. The study was performed according to the principles of the Declaration of Helsinki, the Clinical Good Practice rules for medical research, and the most updated privacy regulations. Participants’ details were kept confidential. All participants provided informed consent prior to completing the questionnaire and were aware that they had the right to withdraw their participation and information at any time during the research.

## Results

### Descriptive analysis of the sample

Of the 39 Emilia-Romagna CMHC directors, 28 gave their consent to participate in the study and were therefore enrolled (response rate: 71.8%). Gender was equally distributed among the sample with 14 males and 14 females (50% of each). Participants had been working as CMHC directors for an average of 7.6 years (standard deviation (SD): 5.6 years, range: 1–21). Five (17.9%) worked in CMHCs of the MHD of Reggio Emilia, 7 (25%) in Modena, 4 (14.3%) in Bologna, 3 (10.7%) in Piacenza, 4 (14.3%) in Parma and 5 (17.9%) in Ferrara. Ferrara had the department with the longest mean duration of service among the respondent CMHC directors (mean: 10 years, SD = 5.39, range: 1–15), while Parma directors had the shortest mean period of service (mean: 5 years; SD = 2.94; range: 1–8).

### Qualitative thematic analysis

The thematic qualitative analysis provided 533 coded segments. They were grouped a posteriori in six thematic macro-areas as shown in the codebook list (Table 1). They were as follows: (1) initial allocation of PMHP (70 segments); (2) request to change the allocated PMHP (195 coded segments); (3) users’ request to choose PMHP (194 segments); (4) relevance of the explored topics (30 segments); (5) perceived usefulness of written policies and guidelines on the explored topics (23 coded segments) and (6) need to involve users in policy-making (21 segments).

**Table 1:** Qualitative analysis. codebook of nodes’ topics and subtopics

Topics and subtopics	No of segments coded
(1) Initial PMHP allocation	70
Criteria	49
First contact with the same PMHP	2
Previous therapeutic relationship with the same PMHP	1
Random allocation (“fixed-rota”)	19
Specific PMHP expertise/interest in the user’s disorder	11
Geographical	11
Requested by GP	2
Workload balance between PMHPs	3
Policies	21
Available	14
Not available	7
(2) PMHP change	194
Management	55
Acceptance of the request	35
Refusal of the request	20
Modality	29
Direct request	6
Indirect request	23
No users requests per year	26
Policies	26
Available	13
Not available	13
Reason	58
Personal dissatisfaction for the PMHP	9
Hope in change of medication	17
Other users’ opinions	8
Personal feeling	9
PMHP specific expertise/interest in the user’s disorder	2
Previous compulsory admission caused by PMHP	10
User/PMHP matching (for gender, age or ethnicity)	3
(3) PMHP choice	195
Management	38
Acceptance of the request	22
Refusal of the request	16
Modality	23
Direct request	7
Indirect request	16
No users’ requests/year	26
Policies	26
Available	4
Not available	22
Reason	40
Requested by GP	3
Other users’ opinions	17
Personal feeling	5
PMHP specific expertise/interest in the user’s disorder	6
Previous therapeutic relationship with the PMHP	1
User/PMHP matching (for gender, age or ethnicity)	8
PMHP request to change the allocated user	2
(4) Relevance of the topic	30
High	6
Average	13
Low	9
None	2
(5) Usefulness of written policies if available	23
Yes	22
No	1
(6) Users’ should be involved in written policy making	21
Yes	19
No	2

**Fig. 1:**
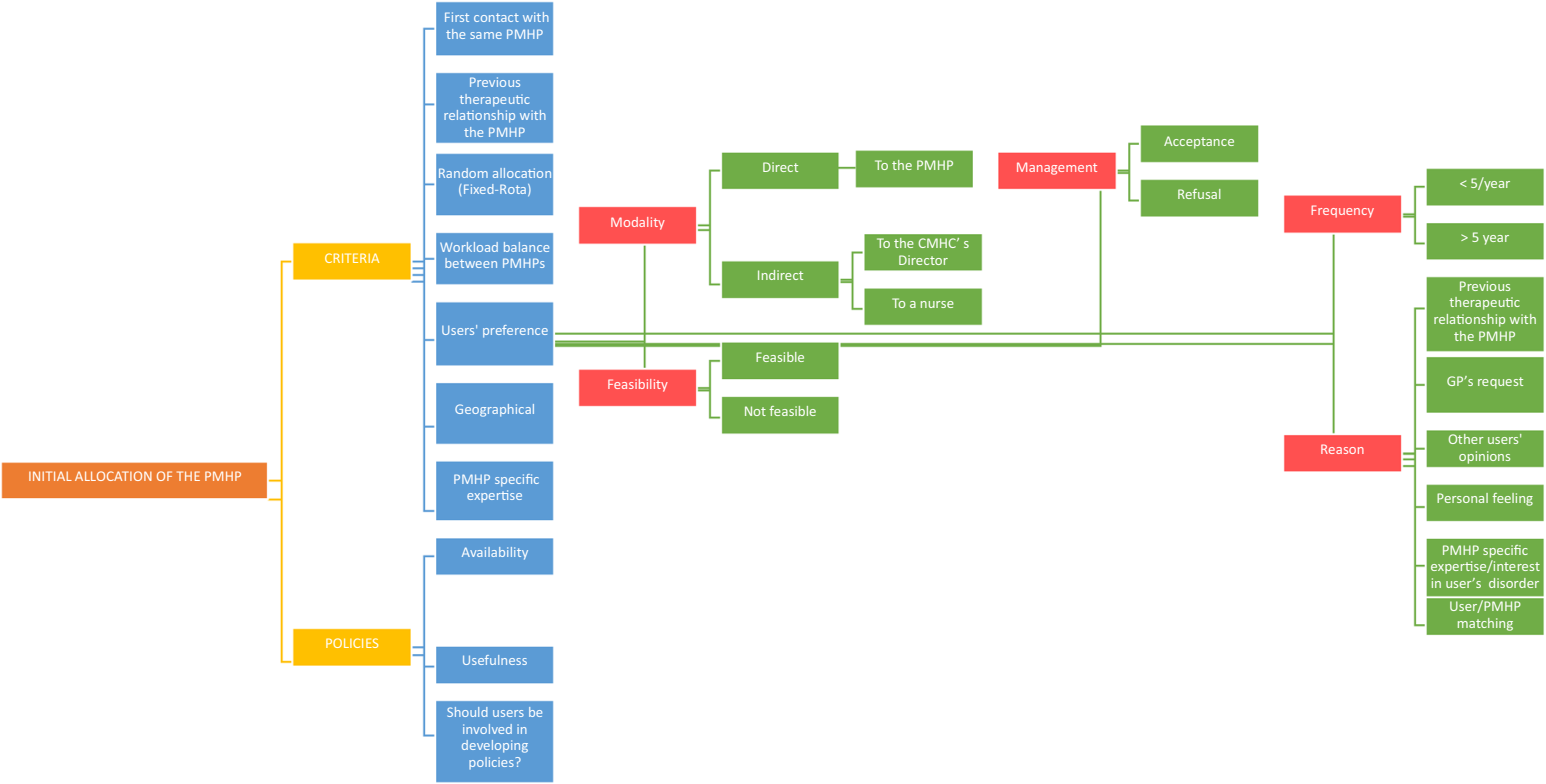
Initial allocation of the PMHP: coding tree of nodes

Figure 1 provides a coding tree of the major and minor explored themes (called “nodes” in Nvivo12 software) on the topic of initial allocation of the PMHP.

**Fig. 2:**
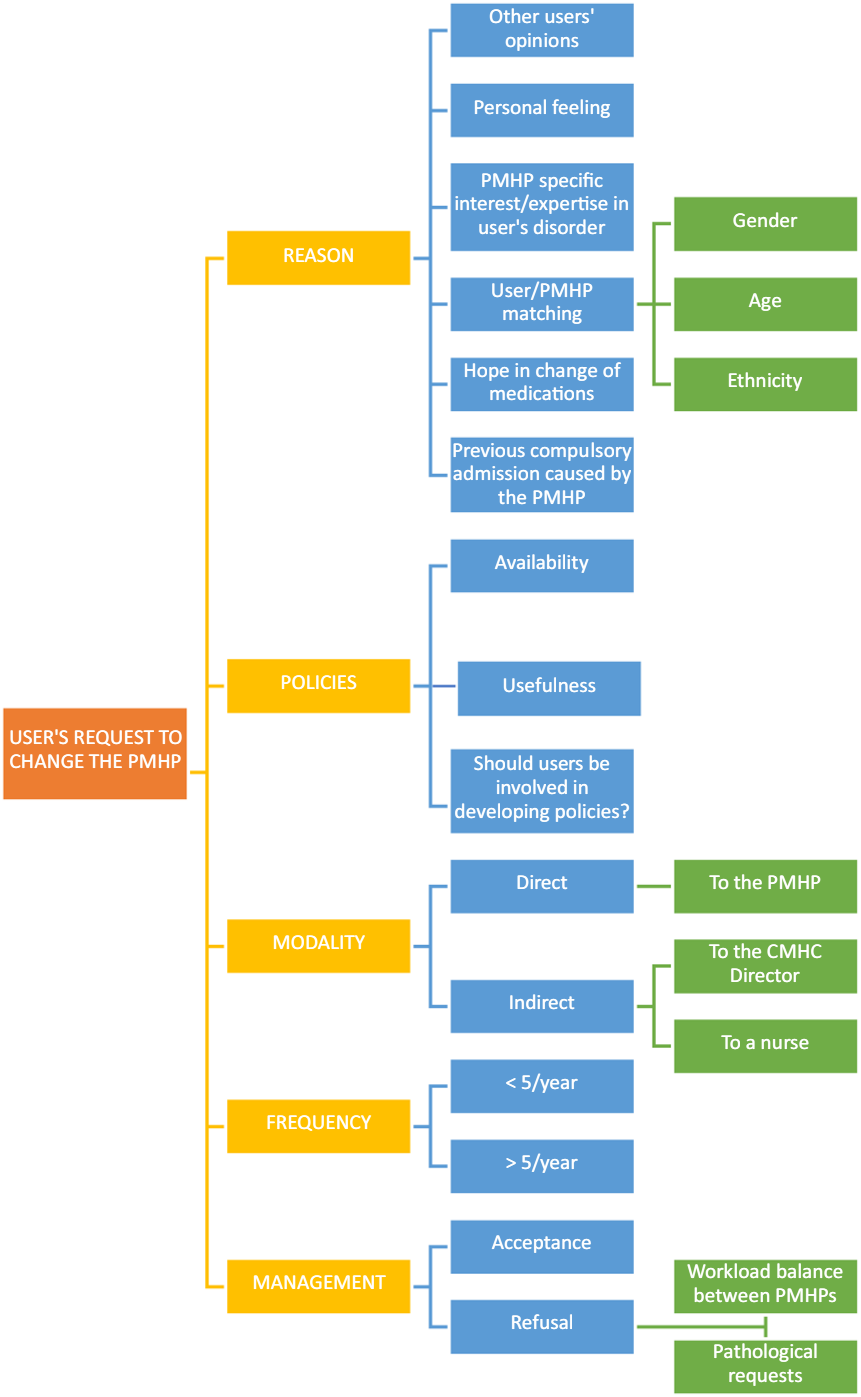
Change of the PMHP: coding tree of nodes

Figure 2 provides a coding tree of the major and minor explored nodes on the topic of request to change the PMHP.

### Quantitative analysis

The results of the quantitative analysis, integrated with the sub-headings derived from the qualitative analysis are displayed in Table 2.

**Table 2:** Quantitative analysis

	N	%
(1) Initial PMHP allocation		
Criteria		
Previous therapeutic relationship with the same PMHP	1	3.6
Random allocation (“fixed-rota”)	11	39.3
Specific PMHP expertise/interest in the user’s disorder	6	21.4
Geographical	6	21.4
Workload balance between PMHPs	4	14.3
(2)PMHP change		
Management		
Acceptance of the majority of the requests	12	42.9
Acceptance of the minority of the requests	13	46.4
Acceptance of all the requests	2	7.1
Refusal of all the requests	1	3.6
Modality		
Direct request	24	85.7
Indirect request	4	14.3
No users requests per year		
0 request	1	3.6
1–5 requests	16	57.1
5–10 requests	8	28.6
10–15 requests	1	3.6
> 15 requests	2	7.1
Policies		
Available	14	50
Not available	14	50
Reason		
Personal dissatisfaction for the PMHP	5	17.8
Hope in change of medication	9	32.2
Other users’ opinions	4	14.3
Personal feeling	3	10.7
PMHP specific expertise/interest in the user’s disorder	1	3.6
Previous compulsory admission caused by PMHP	5	17.8
User/PMHP matching (for gender, age or ethnicity)	1	3.6
(3) PMHP choice		
Management		
Acceptance of all the requests	3	10.7
Acceptance of the majority of the requests	8	28.6
Acceptance of the minority of the requests	13	46.4
Refusal of all the requests	4	14.3
No users’ requests/year		
0	5	17.8
1–5	11	39.3
5–10	4	14.3
10–15	4	14.3
> 15	4	14.3
Policies		
Available	15	53.6
Not available	13	46.4
Reason		
Requested by GP	2	7.1
Other users’ opinions	12	42.9
Personal feeling	4	14.3
PMHP specific expertise/interest in the user’s disorder	3	10.7
Previous therapeutic relationship with the PMHP	1	3.6
User/PMHP matching (for gender, age or ethnicity)	6	21.4
(4) Relevance of the topic		
High	3	10.7
Average	15	53.6
Low	8	28.6
None	2	7.1
(5) Users’ should be involved in written policy making		
Yes	20	71.4
No	8	28.6

1. Initial allocation of the PMHP

Concerning the most common criteria for users’ initial allocation to a PMHP, according to participants, casual allocation by fixed rotation is commonly performed (39.3% of cases), followed by matching the user to a PMHP with specific expertise/interest in the user’s disorder or as per a geographical catchment area rule (21.4% each).

2. Users’ request to change the allocated PMHP

In the last 12 months, 16 CMHC directors, (57.1% of the respondents) received one to five users’ requests to change their allocated PMHP, 8 (28.6%) received five to 10 requests, one (3.6%) received 10 to 15 requests, two (7.1%) had more than 15 requests per year and one (3.6% of the sample) received no requests at all.

According to CMHC directors, the reasons for users asking for a change in the allocated PMHP were mostly hope in drug therapy changes (32.2%), negative feelings after being compulsorily admitted (17.8%), personal dissatisfaction with the PMHP (17.8%), other users’ opinions (14.3%), personal feelings (10.7%), need for a better/different user/PMHP match (in terms of gender, age or ethnicity) (3.6%) and PMHP’s specific expertise/interest in the user’s disorder (3.6%).

“Sometimes, behind users’ requests there could be the attempt to change the drug therapy, so the psychiatrists should never forget to explore this topic with their patients.” (A CMHC director)

In most cases (85.7%), users addressed their request to change the PMHP not to the PMHP him/herself but to the CMHC Director or a nurse; only a minority of cases (14.3%) were reported to have directly expressed their desire to change to their PMHP.

“In most cases, users express their requests to their nurse because they feel free to say what they think.” (A CMHC director)

However, 46.4% of CMHC directors reported accepting only some of the users’ requests they received in the 12 months prior to filling out the questionnaire, 42.9% admitted accepting the majority of users’ requests, 7.1% accepted all requests and 3.6% did not accept any.

Finally, three participants outlined that sometimes the request to change may come not from the user, but from the PMHP, especially in case of long-lasting therapeutic relationships.

“I believe that in cases of particular incompatibility even the doctor should be able to ask not to follow a user anymore.” (A CMHC director)

3. Users’ request to choose the PMHP

In the last 12 months, 11 CMHC Directors (39.3% of the total sample) received one to five requests from new users to choose their PMHP from the start. On the other hand, four directors (14.3%) received 5 to 10 requests, four (14.3%) had 10 to 15 requests, four (14.3%) had more than 15 requests for year, and five (17.8%) received no requests at all.

Users’ common reasons behind asking to choose their PMHP from the start were as follows: other users’ opinions (42.9%), user/PMHP matching for age, gender or ethnicity (21.4%), personal feelings (14.3%), PMHP’s specific expertise/interest in the user’s disorder (10.7%), request from the GP (7.1%) and previous therapeutic relationship with the PMHP (3.6%).

However, 46.4% of the CMHC directors accepted only some of the users’ requests to choose their PMHP from the start in the previous 12 months, 28.6% accepted most of the users’ requests, 10.7% accepted all requests and 14.3% did not accept any.

Moreover, in the qualitative analysis, respondents highlighted organisational and practical issues, supporting the system as it is (the “fixed-rota” system described above), which serves the aim to distribute caseloads more equally across professionals.

“There is a need for a fair distribution of workload among professionals of equal competence…” (A CMHC director)

4. Relevance of the topics

The topics addressed were felt to be relevant by the majority of participants (N = 15, 53.6%), highly relevant for 3 subjects (10.7%), of little relevance for 8 (28.6%) and of no relevance for 2 (7.1%).

5. Written policies on the explored topics

Written guidance on how to deal with users’ request to choose the PMHP was available according to 53.6% of respondents, while guidance on changing the PMHP was available in 50% of the CMHC. Two MHDs (Parma and Bologna) implemented a list of operative instructions dedicated to such requests, which essentially consist of submitting the request to CMHC director and subsequent discussion with relatives (when possible/relevant) and within the CMHC team.

6. Involvement of users in policy-making

According to 71.4% of respondents, users should be involved in the co-production of written policies regulating the topics discussed here.

## Discussion

This study explored the opinions and existing policies on how to deal with requests from users of mental health services to be more involved in the choice or to change their allocated PMHP by surveying directors of services.

A random allocation of users to PMHPs (so-called “fixed-rota”) is by far the most common method; according to such a method, users, as well as professionals, do not generally have a say in the initial match user/PMHP. This finding corresponds with results of our focus group based qualitative study on the same topic that was conducted in Modena in 2017 [25] and with results of other studies on this topic [20, 22].

In general, there was agreement among participants on considering the topic of the choice and change of PMHP as relevant and requiring discussion. This finding is also in line with previous studies [24, 29]. Interestingly enough, all respondent CMHC directors regularly deal with users’ requests for specific initial allocation to a particular psychiatrist and for change of PMHP. Further, the CMHCs that have specific operative instructions on the issue involve directors. This may reflect the sensitivity of the topic, at least in Italy; a request for change is often perceived by the treating professional as a negative judgment by the user. Additionally, the authors feel that the need to involve the director in such decisions reflects the desire to apply a higher threshold to the change, which is seen as something to be discouraged and often interpreted as a groundless complication of the “standard” system.

Moreover, the hope that a new psychiatrist will change the prescribed psychotropic medications was commonly recognised as the main motivation prompting a request to change the PMHP. This result is in line with our previous findings [25], but no other scientific analysis on this topic has been performed so far to our knowledge. It is logical to think that if shared decisions about medication were better implemented, disagreement about medication regimen between users and psychiatrists would be a less likely reason for requesting a change of PMHP. Interestingly, the centrality of disagreements over prescribing as the main reason for requesting change of PMHP may point to a specific risk for services where PMHPs are psychiatrists, as it is generally in Italy; that is, the perception that the key role of mental health services is to dispense medication. This may be exacerbated by the scarcity of alternative treatment and support options, made worse by current staff shortages. This is consistent with the recent finding by Starace et al. who found that the rate of individuals prescribed antipsychotic drugs in Italian mental health services was inversely associated with the rate of mental health professionals available in Italian regions [30].

A previous involuntary admission was another common reason to ask for changing the allocated PMHP, if he/she was the one who took this decision; this finding is consistent with previous studies, highlighting the highly traumatic impact of compulsory admissions on users [31, 32]. Finally, general dissatisfaction or conflict within the therapeutic relationship with the PMHP were identified as frequent motivations for users to ask for a change of their PMHP. Similar findings are also reported elsewhere [33].

Age, gender and ethnicity matching between the user and PMHP were infrequent motivations for requesting a specific PMHP or a change in PMHP, according to CMHC directors’ working experience. This result is line with previous studies investigating users’ gender preferences for their PMHPs [34, 35] but is in contrast with a meta-analysis of 52 studies [36] that showed a moderately strong preference for a therapist of one’s own ethnicity in mental health settings. Nevertheless, it must be noticed that the highest preference was detected among African-American participants, thus limiting the comparison with the population selected for the present research.

As highlighted by three participants, the request to change may come not only from users but also from PMHPs sometimes. This was also found during the aforementioned focus group-based qualitative study on the same topic [18]. Moreover, such requests seem to be more common in the case of long-lasting therapeutic relationships, when the relationship may have reached a critical point [37].

However, quite surprisingly, users’ initial allocation to a PMHP is commonly regulated by unwritten operative instructions in the majority of CMHCs, and the management of users’ requests to choose and/or to change their allocated PMHP is not generally officially codified. Only two MHDs (Departments of Bologna and Parma) have locally implemented written instructions guiding the management of users’ requests to choose and/or change their PMHP, which highlights the relevance of the explored topics.

### Limitations of the study

A few limitations—mostly related to the research methodology—in the present research should be acknowledged.

First, although the process of allocating and changing PMHPs in CMHCs was explored in order to address the level of service users’ involvement, we did not interview service users in this study. However, this was the object of a specific focus group study conducted by the authors that will be reported elsewhere [25]. Second, the present survey was based on an electronic questionnaire; respondents were asked to answer the questionnaire, which was sent by e-mail. However, while low response rates are known to be one of the main shortcomings of surveys diffused via mail or e-mail, the response rate for this research was good. Further, this method of enrolment may also have encouraged replies from younger respondents who may have been more comfortable with electronic-based surveys; nevertheless, nowadays, heads of clinics and centres are usually familiar enough with electronic communication despite their age as it is very commonly used in daily practice.

Third, transcripts were not returned to participants for comments or corrections; however, as several papers on member checks in qualitative research underlines, this limitation is unlikely to significantly affect research findings [38, 39].

## Conclusions

According to our results, neither users nor professionals are generally involved in the initial choice of PMHPs in Emilia-Romagna CMHCs. However, further national-level studies could be implemented to verify the consistency of our results and assess the availability of ongoing policies in different areas. The co-production with users of written criteria or policies for managing users’ requests to choose/change PHMPs could be useful in clinical practice and address an unmet need for the provision of mental health care. This may also lead to a decrease in the perceived coercion in the process of care. Given that coercion in community treatments is not only traumatic, but there is no indication for effectiveness, this seems an important goal to be tested in future research.

## Data Availability

The dataset used and/or analysed during the current study are available from the corresponding author on reasonable request.

## Abbreviations

PMHP: Primary mental health professional
CMHC: Community Mental Health Centre
MHD: Mental Health Department
GP: General practitioner
SD: Standard deviation
